# Evaluating employability in contexts of change: validation of a scale

**DOI:** 10.3389/fpsyg.2023.1150008

**Published:** 2023-08-01

**Authors:** Pilar González-Navarro, Ana Isabel Córdoba-Iñesta, Ana María Casino-García, Lucía Inmaculada Llinares-Insa

**Affiliations:** ^1^Department of Social Psychology, IDOCAL, Universitat de València, Valencia, Spain; ^2^Department of Developmental and Educational Psychology, Universitat de València, Valencia, Spain; ^3^Department of Inclusive Education and Social-Community Development, Universidad Católica de Valencia San Vicente Mártir, Valencia, Spain; ^4^Department of Social Psychology, Universitat de València, Valencia, Spain

**Keywords:** employability, employment, soft skills, labor competences, scale development, human resource management

## Abstract

**Introduction:**

Employability is a crucial factor in managing to emerge and changing job demands. This study validates an expanded version of the Employability Appraisal Scale: EAS-60, as an instrument to identify and improve competencies for employability.

**Method:**

The EAS-60 was tested in a cross-sectional study in a Spanish population. An exploratory study was carried out using a sample of 188 workers, and the scale’s structure was analyzed and confirmed in two Confirmatory Factor Analyses using a sample of 527 workers. Finally, reliability and validity were evaluated.

**Results:**

Exploratory and confirmatory analyses provide evidence supporting the multi-dimensional structure. The scale presents good psychometric properties and criteria for interpreting the scores.

**Discussion:**

The EAS-60 is a reliable and valid instrument. It allows Human Resource Managers to offer career plans at work that include specific actions of job socialization, training, improvement of specific skills, etc. Furthermore, employees can increase their employability and develop their professional careers.

## 1. Introduction

Employability is a meta-competence that integrates different transversal competences that make it possible to be active in the labor market (Llinares-Insa et al., 2020). This approach addresses the “knowing how,” “knowing why,” and “knowing whom” (Eby et al., 2003) of employability, which can be applied to the whole population and are useful for individuals and organizations. Some instruments have been created to assess employability, but we have not found any statistically validated instruments that measure a sufficient number of core competences of employability. Thus, for example, some instruments assess the employability of very specific groups (Sala-Roca et al., 2020), whereas others focus on individual employability factors (López-Miguens et al., 2021) or a future perspective of employability (Gunawan et al., 2019), etc. Many of the scales that measure employability have not been validated (e.g., Brouwers et al., 2015). Moreover, employability scales sometimes do not have a clear theoretical framework (Senan and Sulphey, 2022). Besides, they do not present a holistic view that includes all the indicators of employability (Neroorkar, 2022).

Therefore, there is a need for a standardized and validated questionnaire. The current paper develops and validates the Employability Appraisal Scale (EAS-60), a tool based on the bio-ecological model of employability (Llinares-Insa et al., 2016, 2020) that focuses on the interaction between individuals, their circumstances, and their context. Therefore, this scale contains the individual competences as well as the personal circumstances that evolve him/her and that can influence considerably the employability, and the context as the labor market. This tool uses a methodology to design and implement employability competences, for both prevention and intervention purposes, in order to achieve the horizontal mobility described by Van der Heijden (2002). This mobility benefits both the employee, who can renew and/or broaden his or her competences, and the organization, where the employee’s professional experience can reach other parts of the organization. In fact, the analysis of competences is one of the central topics in the improvement of human resource management methodology (Catalano et al., 2004). Competences are defined as a known and evaluable set of capabilities that allow adequate performance in a real work situation. Thus, competences are key elements in human resource management practice because they involve the ability to mobilize knowledge and practices and reflect on action. It is important to deal with global changes and challenges in order to have an employable workforce with updated employability competences (Van Harten and Vermeeren, 2021). Therefore, the objective of this study is to develop and validate the Employability Appraisal Scale (EAS-60) as an instrument for identifying and locating competences for employability.

## 2. Literature review

### 2.1. Theoretical framework of employability

The term employability has long focused on individuals as the agents responsible for the development of the skills that made them employable workers (e.g., Fugate et al., 2004). From a proactive and sustainable perspective (e.g., Rothwell and Arnold, 2007), the focus of employability changed to meet the challenges of the labor market. Today, employability highlights the concept of competences in its definition, giving particular importance to self-knowledge, managing one’s own career path, flexibility, and adaptability to the context, with an eye on the concept of sustainability and the well-being of individuals (see Di Fabio, 2017). The theoretical frameworks for employability have also evolved, and, as Williams et al. (2019) state, different stakeholders highlight different approaches to employability.

In a systematic review of the literature on employability, Williams et al. (2016) identified 16 different conceptualizations and pointed out the need to combine a variety of factors (such as job market demand, career management, capital, demographic components, etc.) to better understand employability and successfully define and measure it. They also highlighted the need to consider these factors within a holistic conceptualization of employability. We agree that an integrative theoretical framework is essential for an effective evaluation of employability. For this purpose, this study is based on the bioecological model of employability (Llinares-Insa et al., 2016, 2020), one of the most integrative theoretical frameworks for studying employability. It incorporates the responsibility of society, companies, and the context into the equation that defines employability. Thus, employability is defined as a social construction that results from the reciprocal interaction between individuals and their environment with regard to the acquisition and maintenance of employment. From this perspective, employability is conceptualized as a meta-competence, and the person is active and intentional in a context that will hinder or facilitate the possibilities for employability. As a meta-competence, employability is an umbrella that integrates a whole set of transversal competences for employment. These competences are the bridge that connects theory and practice, that is, the manifestation of applied theory, as Bach and Sulikova (2019) pointed out.

### 2.2. Employability scales

To analyze the existing scales to assess employability, we carried out a review in scientific databases. Most of them only use samples of students, or they are designed to assess the employability of students (e.g., Senan and Sulphey, 2022). Some scales are created to evaluate employability in very specific contexts or samples, such as the “Athlete Competency Questionnaire for Employability” (Smismans et al., 2021) or employability of millennials in Mexico (De la Garza et al., 2020), employability of Italian workers, and employability as the perception of the probability of getting a new job (Lodi et al., 2020). Some studies analyze the antecedents of employability (e.g., innovative skills and abilities, along with knowledge, personality attributes, career-related traits, emotional intelligence, and perception of efficacy), as in the case of “The scale of Employability and innovation” (Singh et al., 2017). Most of these scales are focused on personal attributes of employability. However, as we argued above, to obtain a holistic conceptualization of employability, it is necessary to include in the equation the reciprocal interaction between individuals and their environment in relation to the acquisition and maintenance of employment.

For this purpose, some researchers, such as Sala-Roca et al. (2020), focus on the employability competences that are transferable to all kinds of jobs. They use the “Situational Test of Basic Employability Competences Development” to evaluate eight basic competences, regardless of the type of work and professional sector. However, they are only interested in socio-educational interventions for adolescents and young people. Some studies use the concept of sustainable employability (e.g., Jabeen et al., 2022; Picco et al., 2022), connecting personal and social resources to meet labor market demands and develop personal potential and aspirations. For example: the Maastricht Instrument for Sustainable Employability (Houkes et al., 2020) focuses on the areas of meaning of sustainable employability, the level of sustainable employability, factors that affect sustainable employability, general responsibility for sustainable employability, and responsibility for the factors that affect sustainable employability. In contrast, Tésits et al. (2021) proposed the concept of territorial employability to highlight the relevance of the characteristics of the labor market and the dominant factors that affect employability (income position, family status and care systems group, mobility, and networking, etc.). Nevertheless, the focus is on disadvantaged people, and it is currently only a proposed theoretical model. Thus, we have not found any validation studies of employability scales with different samples and results that can be generalized or studies with scales that can be used for any population, context, or career development. In Di Fabio (2017) review of empirical studies on employability the author emphasizes the need to a more holistic, comprehensive instrument analyzing every parameter of employability, both subjective and objective, in order to highlight the complexity of this construct. However, five years later, in Neroorkar (2022) review since 2000 until 2022 still points out to the same gap. This gap makes it difficult to advance the knowledge about employability because we cannot identify standard levels of soft competences in employability for the general population. Knowing this standard level would allow us to identify the upper and lower limits of any specific competence to improve employability. In addition, as Alcover et al. (2021) stated, when the measurement criteria are not unified, solid conclusions cannot be drawn about the relevance and use of the results.

In this context, one exception is the Employability Appraisal Scale (EAS) (Llinares-Insa et al., 2018), based on the Bioecological Model by Bronfenbrenner and Morris (2006), which uses a system of standard job competences following the recommendations of Catalano et al. (2004). We used these competences as a framework to develop the EAS-60, an instrument designed to evaluate the transversal employability competences and generate individualized training itineraries adapted to the particular social context. The EAS contains five employability indicators (resources and strengths for employment, risks and weaknesses for employment, self-control, proactive behavior, and self- presentation skills) that are the starting point for the development of the transversal competences of the EAS60. We hypothesized that the new scale will contain these five employability indicators as well as the EAS-60 is created from EAS and tries to show a holistic and more complete scale (with a wider range of items in order to better cover the core competences involved on the process of employability) sensitive to the contextual changes on employability and the labor market.

### 2.3. Employability evaluated by the EAS-60 as a methodology for human resource management

A system of job competency standards has important benefits for the company, employees, employers, and society in general (Catalano et al., 2004). For companies, obtaining objective information through competences helps internal human resource management by reducing hiring costs and increasing productivity and general competitiveness. In addition, this information contributes to the well-being of workers because it reduces occupational risks (e.g., the possibility of accidents, burnout, etc.) due to inadequate training. For workers, a competency system goes beyond formal education or training and makes it possible to objectify their knowledge and skills, which allows them to increase their employability and labor mobility. Moreover, it can help with career development. For society, a system of competency standards makes it possible to clarify the link between the skills required by employers and the education and training workers receive.

Managing organizational change in a globalized world requires human resource management (Roscoe et al., 2019). Thus, it is currently called green human resource management. Hence, new fields of study emerge for human resources action and intervention (see Ren et al., 2018). In fact, as Chiappetta Jabbour et al. (2019) point out, human resources management is what will lead to organizational sustainability and address the “human side” of organizations.

Human resource management theory should help companies adapt to changes in the economic, social, and technological environment. We want to contribute to this through the methodology offered by the EAS-60. The EAS-60 is a sensitive to environmental changes. It is a tool that can be used to identify and locate skills for employability, create training itineraries to improve employability, and develop a career plan that includes specific actions of labor socialization, training, and improvement of specific competences, etc. It is designed to be applicable to all types of labor and social differences and different individuals and/or groups, whether young university students or adults in a work integration social enterprise. Furthermore, being able to evaluate all the competencies that are evaluated through the EAS-60 could help to mitigate what Climent-Rodríguez et al. (2019) call the “grief” suffered by older people in their job search due to the negative association between age and assessment of possibilities of return to work.

In addition, as Marcus and Fremeth (2009) suggested, Green Human Resources Management aims to satisfy stakeholders, and the cornerstone is the harmonious interconnection between the economic, social, and environmental spheres, the so-called “triple bottom line” (Elkington, 1994). The EAS-60 also addresses this need for an assessment tool within the theoretical framework of the bioecological model of employability (Llinares-Insa et al., 2016, 2020), given that it is sensitive to variations in employability resulting from this dynamic interconnection among the three spheres of employability (person-social-environment). Finally, the EAS-60 is a short, easy to complete, and agile instrument that takes into account and minimizes the risks of social desirability. Therefore, this study develops and validates the Employability Appraisal Scale (EAS-60) as a tool for identifying and locating competences for employability.

## 3. Method

### 3.1. Participants and procedure

Data were collected through a self-administered online questionnaire. Subjects’ participation was voluntary and anonymous. This study was carried out with the approval of the Ethics Committee of the University of Valencia (UV-INV_ETICA-1571107) and is in accordance with the ethical recommendations of the Committee on Publication Ethics (COPE).

Participants in Sample 1 were 188 Spanish workers in different organizations, unemployed people, and graduates. The gender distribution was 59.3% female and 40.7% male, with a mean age of 30.34 years (SD = 10.34), ranging from 19 to 63 years. In terms of their education level, the majority had university degrees (59.3%), followed by university master’s degrees (14.8%) and higher vocational training (11.9%). Additionally, 31.1% were not working at the time.

The participants in Sample 2 were 527 Spanish workers in different industrial, commercial, and service organizations, unemployed people, and graduates. More women (69.1%) participated than men (28.3%), with a mean age of 36.07 years (SD = 14.28), ranging from 18 to 64 years., The majority had a university degree (48.6%), followed by university master’s degrees (17.6%), vocational training (16.1%), and bachelor’s degrees (13.3%). Moreover, 43.9% were not working at the time. The total sample was a convenience sample with an adequate sample size (Tabachnick and Fidell, 2007).

### 3.2. Measures

Employability was measured with the EAS-60, which contained 60 items (Supplementary material), described below.

*Self-Efficacy* was assessed with an 8-item factor, based on the Spanish version of the New General Self-Efficacy Scale (Chen et al., 2001). Responses were given on a 5-point Likert-type scale (ranging from “Strongly disagree”-1 to “Strongly agree”-5) (“I achieve what I set out to do “). Cronbach’s alpha was 0.91.

*Resilience* was assessed with the Spanish version of the Resilience Scale by Connor-Davidson (CR- RISC 10) (Soler et al., 2016), which is composed of 10 items. A sample item is “I can handle any situation.” Items were scored on a 5-point Likert scale (from 1-Strongly disagree to 5-Strongly agree). Cronbach’s alpha was 0.89.

### 3.3. Item generation

Following the recommendations of Simms and Watson (2007), we developed and validated the EAS- 60 scale. In the first phase of the study, we carried out a review of the literature on scales that measure employability. We used the recommendations for scale construction by Wright et al. (2017) and Diamantopoulos and Winklhofer (2001). The aim of the first study is to improve and expand the EAS. We expect the same multi-dimensional structure for this scale as for the EAS, and that it will be applicable to all social groups.

To write the scale items, we followed the conventional guidelines regarding: clarity, difficulty, length, directionality, lack of ambiguity, grade appropriate language and context, avoidance of jargon and trick items, and unanimous author endorsements (e.g., Haladyna et al., 2002), a five-point Likert format was used for scoring, following Lloret et al. (2014). We wrote 4 or 5 items for each assessed competency.

Next, to verify the content validity and applicability of the first version, two processes were carried out. In the first one, we conducted five focus group sessions with three and/or four participants. The participants were two doctors in work and organizational social psychology, one social psychologist specializing in social vulnerability, and one social psychologist specializing in employment services. Based on Van Wingerden and Niks (2017), this group analyzed: (a) whether each item could be understood by the different working populations at any level of education and Spanish language proficiency and (b) the relevance of the item for assessing employability. Then, once the second version of the questionnaire had been created, we asked four professionals to evaluate the same questions qualitatively. This multidisciplinary team of judges consisted of education professionals, education and developmental psychologists, social psychologists, and social workers. Based on their observations, we elaborated the second version of the measurement instrument with 60 items. Afterwards, we performed a pilot test in a small convenience sample (*n* = 70). This sample was composed of workers, unemployed young people, and volunteer university students. We asked them to fill out the questionnaire and add their observations, criticisms, and suggestions. Their answers allowed us to assess the effectiveness and relevance of the questionnaire, the difficulty of understanding some words, possible ambiguities, missing information, or the adequacy of the length. Next, we elaborated the fourth version of the measurement instrument with 60 items. Finally, we prepared an introduction that contained the instructions for filling out the questionnaire, guaranteeing anonymity and voluntary participation and acknowledging their collaboration in the research. Moreover, we included answers about socio-demographic, labor, and health issues. Thus, we obtained the final version of the scale: the Employability appraisal Scale-60 (EAS-60).

Content validity was tested by performing the following actions. A review of the employability literature was conducted. In addition, a theoretical framework was established that defines the topic of employability, its constituent elements, and its relationships with other constructs. In addition, a group of experts evaluated the adequacy, adaptation, and/or translation of each competency, indicator, and item. During the process of re-specifying the items, we ensured that the items remaining in the questionnaire conceptually covered the full scope of each competency assessed and each indicator, as well as the entire concept of employability as a whole. Thus, the items covered the entire scope of the latent variable.

### 3.4. Data analysis

First, we examined the factor structure of the scale. An Exploratory Factor Analysis (EFA) was performed. Barlett’s test and the Kaiser-Meyer-Olkin (KMO) were used to assess the adequacy of the analysis for structure identification and sampling adequacy (Bartlett, 1954). A score ≥ 0.7 indicates that the EFA is appropriate (Kaiser, 1974). When the KMO value is <0.05, it is unacceptable (Tabachnick and Fidell, 2007). Then, we calculated the EFA with Unweighted least-squares factor extraction (LS) and Oblimin rotation to maximize the variance between factors with Kaiser normalization to identify meaningful components (Lloret et al., 2014). The number of latent factors was determined by using the theoretical framework that defines the construct (Step 1) and the number of factors that were extracted from the EAS (Llinares-Insa et al., 2018). For the extraction of the EAS-60, we used the Kaiser rule (eigenvalues greater than two) (Kaiser, 1960) and scree cut-off points (Cattell, 1966). All extraction communalities were restricted to ≥0.3 (Stevens, 1992; Field, 2009). Cronbach’s alpha and Composite Reliability (CR) were used to analyze internal consistency. A score ≥ 0.6 indicates good reliability (Fornell and Bookstein, 1982).

Second, we analyzed the factorial structure of the EAS based on the results obtained in the EFA (Jöreskog and Sörbom, 2006). We started by computing the polychoric correlation matrices among the items. Subsequently, two Confirmatory Factor Analyzes (CFA) were carried out; the first CFA analyzed the scale structured in five factors. The second CFA was performed to test a single-factor structure. Later, we used Multigroup Confirmatory Factor Analysis. To assess model fit, we used absolute (Jöreskog and Sörbom, 2006) and relative (Marsh et al., 1996) indices: a) *χ*^2^ statistic and *χ*^2^/df < 5 (Hooper et al., 2008), b) the Googness of fit index (GFI) and the Adjusted Goodness of Fit Index (AGFI), with cut-off criteria of 0.90 or higher (Hu and Bentler, 1999), and c) the Root Mean Square Error of Approximation (RMSEA) and Root Mean Square Residual, with values of 0.08 or lower indicating good fit (Hair et al., 2006).

Third, drawing on Fornell and Bookstein (1982), to evaluate the psychometric proprieties (reliability and criterion validity), we calculated the average variance extracted (AVE). Thus, we evaluated discriminant validity. To analyze concurrent and external validity, we correlated employability with self-efficacy and resilience using Pearson’s product–moment correlation coefficients. Some studies have confirmed the direct relationship between resilience and employability (Semeijn et al., 2019) and between self-efficacy and employability (Berntson et al., 2008). Moreover, Tentama and Zulfikar (2021) found that self-efficacy is a key variable in increasing employability. Based on Cohen (1988), values below 0.30 indicate a relationship with a small magnitude, coefficients greater than 0.3 indicate a moderate magnitude, and values greater than 0.50 indicate a large magnitude.

Fourth, using the scores for each factor, the rules for interpreting the results were established. For this purpose, we requested information on percentiles. To establish the statistical norms, we adapted the proposal of Schaufeli and Bakker (2003). We established five categories: (a) very low: <10th percentile, (b) low: ≥10th percentile to <25th percentile, (c) medium-low: ≥25th percentile to <50th percentile, (d) medium-high: ≥50th percentile to <75th percentile, (e) high: ≥75th percentile to <90th percentile, and (f) very high: ≥90th percentile. We used SPSS 26 and EQS 6.1 software to conduct the statistical analyzes. There have been no missing values and not removed outliers.

## 4. Results

First, we calculated the descriptive statistics (means and standard deviations) for the items. Skewness and kurtosis values showed that there was deviation from the normal distribution (not lie −1/1). Then, we calculated the correlation values between the total item-scale and the subscales, and they were generally adequate.

Second, to analyze the dimensionality of the scale, we used Sample 1. The results of the Kaiser- Meyer-Olkin test showed that the data were suitable for factor analysis (*χ*^2^ = 4291.47, df = 1770; *p* < 0.001 on Bartlett’s sphericity test and value = 0.75). Moreover, the EFA showed a very consistent internal structure with good fit (mean communalities extracted ≥0.5), and the amount of variance explained was 36.55%. Five factors were obtained with factor loadings ≥0.30 and alpha factors ≥0.60. The results of the first analysis allowed us to keep the original 60 items on the scale and structure them into five factors. Factor 1, resources and strengths for employment, refers to behaviors of persistence, autonomy, initiative, planning, and organization that favor the search for a job and maintenance and progress in the job market insertion process. Factor 2, risks and weaknesses for employment, refers to the lack of individual aspects of a personal nature (especially time management skills), along with the awareness of deficiencies in objective qualifications required for work in any area (training and previous work experience). Together, they express factors that would make labor insertion and maintenance more difficult. Factor 3, self-control, refers to control of impulses and negative emotions (anger, frustration, annoyance) that have to be managed in any interpersonal relationship, including relationships in the work area with co-workers, customers, bosses, managers, or subordinates. Factor 4, proactive behavior, refers to continuous learning and progressive improvement and thriving in the personal and work context, despite the obstacles that might arise. Factor 5, self-presentation skills, refers to behaviors aimed at personal care and the image we show in the working world. Some of them were directly related to active job search behavior and others to skill requirements, external appearance, and professional updating.

The next step in the validation was to carry out the CFA. For this purpose, we used Sample 2. Descriptive analysis was performed, and the scores for the skewness and kurtosis coefficients showed no similarity to the normal curve because the coefficients were not close to zero and < 2.0. The Kaiser-Meyer-Olkin measure was statistically significant (*p* < 0.001 on Bartlett’s sphericity test), with a 320 value of 0.90 (≥0.5).

In the CFA, we used an inter-correlated five-factor model (see Table 1), which agrees with the results of the EFA study, and a one-factor model. The *χ*^2^/df values of the two models (one-factor and five- factor) were < 5.00 (Table 1), which indicated an adequate model fit. Moreover, the goodness-of-fit indices of the two models showed that both structures were acceptable. However, the GFI and AGFI indices for the one-factor model did not reach 0.90, although they were near it. According to Baumgartner and Homburg (1996), these indexes indicate an acceptable fit if they are ≥0.8. Moreover, the Wald Test of the five-factor model indicated the importance of changing Item 60 to Factor 5. The results are presented in Table 2. With this specification, the estimation of the two models showed good overall fit. Next, we estimated the correlations among the five factors in the re- specified model, which were about 0.3 (*ρ* = 0.001). Then, we compared the chi-squared test of the two models (one/ re-specified five-factor model). It was statistically significant (Δ*χ*^2^ = 1682.816, Δdf = 16.82, *ρ* < 0.01), which indicated that the five-factor model was the most parsimonious model. However, it also indicated that employability can be analyzed as a single-factor construct.

**Table 1:** Goodness of fit index for confirmatory factor analysis of the four models.

	*χ* ^2^	dg	*χ*^2^/dg	GFI	AGFI	RMSEA	RMR
Single-Factor Model	7366.98	1,710	4.30	0.88	0.87	0.07	0.08
Five-Factor Model	5681.31	1,700	3.34	0.91	0.91	0.05	0.07
Respecified Five-Factor Model	5684.16	1,700	3.34	0.92	0.91	0.05	0.07
Multi-sample	5684.16	1,700	3.34	0.92	0.91	0.05	0.07

*χ*^2^, chi-square; df, degrees of freedom; GFI, Goodness of Fit Index; AGFI, Adjusted Goodness of Fit Index; RMSEA, Root Mean Square Error of Approximation; RMR, Root Mean Square Residual.

**Table 2:** Correlations between study variables.

	Self-efficacy	Resilience
F1. Resources and strengths	0.59**	0.60**
F2. Risks and weaknesses	−0.28**	−0.36**
F3. Self-control	0.45**	0.44**
F4. Proactive behavior	0.24**	0.34**
F5. Self-presentation skills	0.42**	0.47**

**p < 0.01; SD, Standard Deviation.

Afterwards, we calculated the Average Variance Extracted (AVE). Then, we correlated employability with self-efficacy and resilience using Pearson product–moment correlation coefficients. To consider discriminant validity, we compared the AVEs and the correlations, following Hair et al. (2006). The results showed that the correlations did not exceed the AVEs of any of the latent constructs.

To analyze the concurrent validity of the EAS, we calculated its relationships with self-efficacy and resilience. The results are shown in Table 2. Correlations were moderate (≤0.40); these correlations were a guarantee of adequate validity (see Table 2). Thus, we can state that there is concurrent and external validity.

The last step consisted of the reliability analysis (internal consistency). The results are shown in Table 3. Here, the Cronbach’s alpha of three of the factors is ≥0.60 for each subscale. However, in Factors 2 and 5, Cronbach’s alpha is ≥0.5 (Table 3). According to Taber, 2018, Cronbach’s alpha coefficient values ≥0.5 are acceptable. Even so, these data were corroborated through two methods: split-half method and L4 of Guttman’s formula. The results are also shown in Table 3, and they confirm that both factors have adequate reliability indices. A fourth method of analyzing the internal consistency of the five factors was to calculate composite reliability (CR), as in Bagozzi and Yi (1988). The results indicate that EAS-60 is a reliable and valid instrument for measuring employability.

**Table 3:** Reliability values of the subscales.

	Composite reliability	Alpha	Omega	Guttman split-half
F1. Resources and strengths	0.918	0.91	0.91	0.88
F2. Risks and weaknesses	0.672	0.53	0.82	0.60
F3. Self-control	0.76	0.75	0.75	0.70
F4. Proactive behavior	0.85	0.85	0.82	0.81
F5. Self-presentation skills	0.60	0.50	0.62	0.60

The last objective of the study was to analyze the scores of the Spanish population and generate the norms for the interpretation of the scale. To this end, we performed the Kolmogorov–Smirnov test. The results were significant at *p* < 0.05. Therefore, we concluded that the data were not normally distributed. Subsequently, we calculated the percentiles (Table 4).

**Table 4:** Percentiles of the EAS-60 subscales for the total sample.

Percentile	F1	F2	F3	F4	F5
10	3.20	2.37	1.25	3.60	2.80
25	3.29	2.62	1.75	3.86	3.00
50	3.45	2.87	2.25	4.20	3.40
75	3.62	3.12	2.75	4.46	3.40
90	3.71	3.33	3.50	4.73	3.80
95	3.92	3.46	3.75	4.80	3.80

F1, Resources and strengths for employment; F2, Risks and weaknesses for employment; F3, Self-control; F4, Proactive behavior; and F5, Self-presentation skills.

## 5. Discussion

The aim of this study was to develop and validate the Employability Appraisal Scale (EAS-60) as an instrument for identifying and developing 16 transversal competences for employability (perseverance, stress tolerance, professional qualification, learning to learn, time management, task management, initiative, will and willingness to work, performance-productivity assessment, autonomy, self-esteem, personal care, social skills, technical skills, work experience, and flexibility). These competences are integrated in the five indicators of employability (resources and strengths for employment, risks and weaknesses for employment, self-control, proactive behavior, and self- presentation skills). This is a comprehensive approach to employability because it combines the elements of individuals and their environment in relation to the acquisition and maintenance of employment.

The study of scale dimensionality showed a good fit, and validation and evidence of the psychometric properties were provided. These findings indicate that the scale is a reliable and valid instrument for measuring employability. Moreover, the results showed empirical support for the validity of the EAS-60 in any group (e.g., worker, unemployed, students, immigrants, etc.). Finally, we proposed the statistical norms (percentiles) for the interpretation of the scores on the scale.

The present paper contributes to the employability literature because EAS-60 is an instrument for the assessment, prevention, and improvement of employability. The EAS-60 is based on the theoretical framework of the Bioecological Model of Employability, which understands that employability is constructed based on socio-historical and contextual factors (Llinares-Insa et al., 2016). The EAS-60 is a tool that may provide some insight into the methodology of human resource management research and the shortcomings of existing measures. It covers the construct as a whole taking into account the main indicators of employability, not only individual but personal circumstances and contextual factors as well (Di Fabio, 2017; Neroorkar, 2022); this entails a more comprehensive and holistic approach, necessary to encompass the complexity of this construct.

Moreover, it is an instrument that is sensitive to variations in employability because its components are clearly defined, which means they can be trained. It also serves as a research element and a personal and institutional reflection related to the development of basic job competencies. In addition, it is short, simple to complete, and agile, and it considers and minimizes the risks of social desirability.

For all these reasons, the assessment of employability competences with the EAS-60 has several theoretical and practical implications that are discussed in the following sections.

### 5.1. Theoretical implications

On the one hand, our study supports a multifactorial conception of employability rather than a one- dimensional structure (e.g., Peeters et al., 2020). There are five major interrelated factors, each with their own identity. Several empirical studies suggest the multidimensionality of employability (e.g., Carrein-Lerouge et al., 2021). However, previous research has proposed its multidimensionality, but only with personal factors (e.g., González-Romá et al., 2018). Instead, our results provide empirical evidence for a multidimensional structure that includes both personal and socio-environmental dimensions in order to improve the assessment of employability. Thus, our study adds new insights to the employability literature.

On the other hand, our study extends previous research because the EAS-60 evaluates the employability of any person in any profession, as a multidimensional meta-competence that focuses on the person and the external environment. Therefore, employability assessed with the EAS-60 is important for unemployed people and for professional development, that is, for job search and maintaining and improving employment. We were able to generate a general measure for the Spanish population and, using the percentile values, interpret the score obtained at any time.

### 5.2. Practical implications

Our findings show some practical implications. First, the EAS-60 could provide a way to build a Knowledge Society, reduce poverty and unemployment, and guide employment and educational policies (ILO, 2017). Second, the introduction of this new measure of employability is necessary because it implies some improvements in the processes of evaluation, guidance, and intervention in job transitions and career development (see, e.g., Holland, 2019). The EAS-60 is an adequate instrument to measure employability because it presents validity, reliability, good psychometric proprieties, and criteria for interpreting the scores. Therefore, social agents or human resources services can use it to diagnose employability and its improvement. Third, considering that the EAS- 60 helps to identify domains of employability, counselors could advise people (e.g., graduates, unemployed people, etc.) about programs and strategies to develop specific domains. Fourth, the EAS-60 can help to address the social demand to avoid discriminating (positively or negatively) against vulnerable groups and eradicate social security actions. Social agents need an employability measure in order to offer a career plan instead of a job: a plan that includes socialization, training courses, and anything that can facilitate the person’s job search (ILO, 2015).

Finally, the EAS-60 is an appropriate instrument for managing human resources and improving job security.

### 5.3. Limitations and suggestions for future research

Our study has several limitations that must be taken into account. First, all the data were collected from the same source (participant self-assessments), which could produce common method bias (Podsakoff et al., 2003). To minimize this bias, various precautions were taken. For example, the anonymity of the respondents was guaranteed. Participants were informed that there were no right or wrong answers and that it was important to answer honestly. The order of the items was balanced. Moreover, the EAS-60 has no ambiguous elements, and so biases due to demand characteristics and social desirability were eliminated. Furthermore, the response scale is not bipolar and provides verbal labels to reduce acquiescence bias (Tourangeau et al., 2000). We also avoided socially desirable responses by framing participation as voluntary and confidential. Future studies could limit these biases by using contextual measures, such as external agents (e.g., WISE help desk technicians, HR staff, etc.), to extract a complete individual employability score. Finally, although the heterogeneity of the sample makes it possible to capture the variability of the construct (adults with different educational levels, different occupations, etc.), it can also affect low scores and correlations on the AVE. Further research should use multigroup analysis and factor invariance.

These limitations notwithstanding, our study also shows several strengths. First, the analysis suggests that the scales are reliable and valid enough to continue to be easily applied in future research. Second, by testing a representative sample of participants with different work and educational statuses, we were able to offer a transversal measure that can facilitate comparisons of different groups. Third, this multi-dimensional approach to employability allowed us to capture the fundamental individual and social dimensions.

## 6. Conclusion

This study develops, validates, and demonstrates the good psychometric properties of the EAS-60, based on the Bioecological Model of Employability. The EAS-60 consists of 60 items that assess employability, organized in five dimensions: resources and strengths for employment, risks and weaknesses for employment, self-control, proactive behavior, and self-presentation skills. Our findings can be used by researchers, human resource services, and social workers to examine the employability of workers/unemployed people and design intervention programs (European Commission, 2016).

Employability and talent are two ways to access the labor market. However, knowing one’s level of employability provides information about talent, and when people develop their talent, they develop their employability as well. As everyone knows, incorporating talent into companies is associated with organizational success (Singh, 2021). Thus, a valid and reliable instrument to assess employability is an advantage for a company that wants to achieve success and promote active policies to develop job matching. Furthermore, it is also an advantage for individuals because they can develop their employability by doing their job and contributing their talent.

## Data availability statement

The raw data supporting the conclusions of this article will be made available by the authors, without undue reservation.

## Ethics statement

The studies involving human participants were reviewed and approved by comité Ético de Investigación en Humanos de la Comisión de Ética en Investigación Experimental de la Universitat de València. The patients/participants provided their written informed consent to participate in this study.

## Author contributions

LIL-I, PG-N, AIC-I, and AMC-G wrote the introduction, carried out the statistical analysis of the validation, wrote the results and discussion, and took into account the different references used in the paper. PGN, LIL-I, and AIC-I reviewed the literature and generated the items. All authors contributed to the article and approved the submitted version.

## Funding

This paper was partially funded by Universitat de Valencia and Catholic University of Valencia “San Vicente Mártir” (Valencia, Spain).

## Conflict of interest

The authors declare that the research was conducted in the absence of any commercial or financial relationships that could be construed as a potential conflict of interest.

## Publisher’s note

All claims expressed in this article are solely those of the authors and do not necessarily represent those of their affiliated organizations, or those of the publisher, the editors and the reviewers. Any product that may be evaluated in this article, or claim that may be made by its manufacturer, is not guaranteed or endorsed by the publisher.
