# Supplementary material for: Evaluating employability in contexts of change: validation of a scale
Source: Front Psychol. 2023 Aug 1;14:1150008. doi: 10.3389/fpsyg.2023.1150008 (PMC10428643; doi:10.3389/fpsyg.2023.1150008)
Supplement: Supplementary file 1 [file Table_1.docx]

Supplementary Material

Evaluating employability in contexts of change: validation of a scale

Pilar González-Navarro, Ana Isabel Córdoba-Iñesta, Ana María Casino-García, Lucía Inmaculada Llinares-Insa^*^

*** Correspondence:** lucia.llinares@uv.es

**Supplementary Table 1.** Factor structure, items on the EAS-60 (Spanish and their English translation), and EFA five-factor model

| **Spanish item** | **English item** |
| --- | --- |
| **F1. Recursos y fortalezas para el empleo (Alpha=0.89)** | **F1. Resources and strengths for employment** |
| 26. Organizo mi tiempo y lo aprovecho al máximo. | 26. I organize my time and make the most of it. |
| 22. (-) Suelo dejar las cosas para el último momento | 22. I usually leave everything until the last minute. |
| 2. Soy persistente y constante. Termino lo que empiezo | 2. I am persistent and constant. I finish what I start. |
| 58. (-) Cuando tengo que hacer algo me cuesta mucho ponerme en marcha | 58. When I have to do something, it is very difficult for me to get going. |
| 19. No logro mis objetivos porque no soy constante y me desanimo | 19. I do not achieve my goals because I am not constant, and I get discouraged. |
| 37. Cuando inicio una tarea me gusta acabarla | 37. When I start a task I like to finish it. |
| 25. Soy una persona responsable de todo lo que hago | 25. I am a responsible person for everything I do. |
| 31. Cuando empiezo una tarea lo primero que hago es organizarme | 31. When I start a task the first thing I do is organize myself. |
| 30. (-) Me cuesta organizarme para hacer las tareas | 30. I find it difficult to organize myself to do tasks. |
| 10. (-) No me apetece hacer las tareas que tengo que hacer | 10. I don't want to do the tasks that I have to do. |
| 18. Soy una persona práctica, sé qué tengo que hacer y lo hago | 18. I am a practical person, I know what I have to do, and I do it. |
| 13. (-) No consigo ser constante cuando realizo tareas largas y difíciles | 13. I can't be constant when doing long and difficult tasks. |
| 40. (-) Me cuesta iniciar una tarea cuando no me apetece | 40. It's hard for me to start a task when I don't want to do it. |
| 41. Soy eficaz en mi trabajo/estudios | 41. I am effective in my work/studies. |
| 6. Cuando decido lo que quiero hacer, lo hago | 6. When I decide what I want to do, I do it. |
| 24. (+) Creo que puedo hacer todas las actividades que deben hacerse todos los días | 24. I think I can do all the activities that need to be done every day. |
| 9. Me adapto y hago nuevas tareas en mi trabajo/ estudios/voluntariado, etc. cuando hace falta | 9. I adapt and do new tasks in my work/studies/volunteering, etc. when it is necessary. |
| 59. (-) Me inclino a pensar que soy un fracaso en todo lo que tiene que ver con el trabajo/estudio(AU=Autoestima) | 59. I usually think that I am a failure in everything that has to do with work/study. |
| 29. Soy capaz de organizar mi trabajo/estudios cuando tengo que hacer algo importante | 29. I can organize my work/study when I have to do something important. |
| 50. En el trabajo/estudios/voluntariado, me siento bien conmigo mismo | 50. At work/studies/volunteering, I feel good about myself. |
| 8. (-) Me aburro haciendo las actividades del día a día | 8. I get bored doing day-to-day activities. |
| 17. (-) Suelo cambiar muchas veces de actividad durante el día sin un fin concreto | 17. I tend to change activities many times during the day without a specific purpose. |
| 23. (-) No soy competente porque no estoy al dia en mi profesión | 23. I am not competent because I am not up to date in my profession. |
| 1. Consigo lo que me propongo | 1. I get what I set out to do. |
| **F2. Riesgos y debilidades para el empleo (Alpha=0.60)** | **F2. Risks and weaknesses for employment** |
| 55. (-) Me falta experiencia para que me contraten o cambiar a un trabajo mejor | 55. I lack the experience to be hire or change to a better job. |
| 21. Necesito mejorar mi formación si quiero trabajar o cambiar de trabajo | 21. I need to improve my training if I want to work or change jobs |
| 56. Tengo experiencia laboral en mi profesión | 56. I have work experience in my profession. |
| 12. (-) No tengo suficiente formación para poder trabajar en lo que quiero | 12. I don't have enough training to be able to work. |
| 32. Mi experiencia laboral me permite hacer bien mi trabajo | 32. My work experience allows me to do my job well. |
| 54. (-) Me falta confianza y seguridad en mí mismo en el trabajo / búsqueda de empleo / estudios | 54. I lack self-confidence at work/studies. |
| 3. Tengo suficiente formación para trabajar | 3. I have enough training to work. |
| 44. (-) Hay otros profesionales mejor preparados que yo para trabajar | 44. There are other professionals better prepared than me to work. |
| 52. (-) No sé cómo buscar trabajo | 52. I don't know how to look for a job. |
| 53. (-) Necesito formarme para estar al día en mi profesión | 53. I need to study to be up to date in my profession. |
| 38. En mi trabajo o para poder trabajar estaría dispuesto/a a desplazarme fuera de mi ciudad | 38. In my job or to be able to work, I would be willing to move outside of my city. |
| 57. Trabajaría en cualquier horario | 57. I would work any schedule. |
| **F3. Autocontrol** (Alpha=0.77) | **F3. Self-control** |
| 11. (-) Me enfado con facilidad | 11. I get angry easily. |
| 20. (-) Tengo mal genio | 20. I have a bad temper. |
| 5. (-) Hay cosas que me enfadan y molestan mucho | 5. Some things annoy and bother me a lot. |
| 4. (-) Me resulta difícil controlar mi ira, rabia, malestar | 4. I find it difficult to control my anger, rage, upset. |
| **F4. Conducta proactiva (Alpha=0.82)** | **F4. Proactive behavior** |
| 35. Cuando quiero algo lo busco por todos los medios que se me ocurren o que me dicen | 35. When I want something I look for it by all the means that come to mind or that they tell me. |
| 15. Aprovecho cualquier oportunidad que me permita aprender algo nuevo | 15. I take advantage of any opportunity that allows me to learn something new. |
| 36. Me implico mucho en lo que hago | 36. I get very involved in what I do. |
| 7. Me gusta aprender cosas nuevas, incluso si se trata de pequeños detalles | 7. I like to learn new things, even if they are small details. |
| 28. Soy capaz de aprender de forma autónoma | 28. I am able to learn autonomously. |
| 33. Soy una persona con iniciativa para comenzar las tareas, tomar decisiones rápidas o buscar soluciones a problemas. | 33. I am a person with the initiative to start tasks, make quick decisions or find solutions to problems. |
| 34. Escucho lo que me dicen antes de hablar | 34. I listen to what they say to me before speaking. |
| 16. Yo veo los cambios como una oportunidad para aprender y no como una dificultad | 16. I see changes as an opportunity to learn and not as a difficulty. |
| 42. Me gusta ser yo quien organizo mi trabajo/estudios | 42. I like to be the one who organize my work/studies. |
| 39. Me expreso de forma que los otros/as me entienden | 39. I express myself in a way that others understand me. |
| 51. En una conversación pregunto lo que no entiendo | 51. In a conversation I ask what I don't understand. |
| 14. Cuando necesito saber algo en mi trabajo/voluntariado/estudio, suelo preguntar o pedir que me enseñen a hacerlo | 14. When I need to know something in my work/volunteer/study, I usually ask to taught me how to do it. |
| 27. Confío en mis opiniones incluso si son diferentes a las de los demás | 27. I trust my opinions even if they are different from others. |
| 43. Es importante para mí hacer las cosas como yo quiero | 43. It is important for me to do things the way I want. |
| 60. Como alternativa de trabajo estaría dispuesto a trabajar online | 60. As a job alternative, I would be willing to work online. |
| **F5. Habilidades de autopresentación (Alpha=0.60)** | **F5. Self-presentation skills** |
| 46. Para mí es importante el aspecto personal para encontrar o cambiar de trabajo | 46. Personal appearance is important to me to find or change jobs. |
| 49. Cuido que mi forma de vestir sea acorde con lo que voy a hacer | 49. I take care of the way I dress is in accordance with what I am going to do. |
| 48. (-) Tengo problemas para expresar lo que pienso y relacionarme con otras personas | 48. I have problems expressing what I think and interacting with other people. |
| 47. Me preocupo por mi aseo y mi higiene personal cuando voy a trabajar/estudiar | 47. I'm worry about my cleanliness and my personal hygiene when I go to work/study. |
| 45. (-) Tengo mala presencia y creo que eso puede influir a la hora de encontrar o cambiar de trabajo | 45. I have a bad appearance, and I think that this can influence when it comes to finding and changing jobs. |

© P.González-Navarro, L.I. Llinares-Insa, A.I. Córdoba-Iñesta y J.J. Zacarés González. EAS-60 is an improved and extended version of the EAS (https://www.frontiersin.org/articles/10.3389/fpsyg.2018.01437/full).
